# Activity Profiles and Physiological Responses of Representative Tag Football Players in Relation to Playing Position and Physical Fitness

**DOI:** 10.1371/journal.pone.0144554

**Published:** 2015-12-07

**Authors:** Luke W. Hogarth, Brendan J. Burkett, Mark R. McKean

**Affiliations:** School of Health and Sport Sciences, Faculty of Science, Health, Education and Engineering, University of the Sunshine Coast, Maroochydore, Queensland, Australia; University of Alabama at Birmingham, UNITED STATES

## Abstract

This study determined the physical fitness, match-activity profiles and physiological responses of representative tag football players and examined the relationship between physical fitness and the match-activity profile. Microtechnology devices and heart rate (HR) chest straps were used to determine the match-activity profiles of sixteen tag football players for five matches during the 2014 Australian National Championships. The relationships between lower body muscular power, straight line running speed and Yo-Yo intermittent recovery test level 2 (Yo-Yo IR2) and the match-activity profile were examined using Pearson’s correlation coefficients. Outside players had greater lower body muscular power (ES = 0.98) and straight line running speed (ES = 1.03–1.18) than inside players, and also covered greater very high-speed running (VHSR) distance/min (ES = 0.67) and reached higher peak running speeds (ES = 0.95) during matches. Inside and outside players performed a similar number of repeated high-intensity effort (RHIE) bouts and reported similar mean and maximum efforts per RHIE bout. However, there were differences between playing positions for mean and maximal RHIE effort durations (ES = 0.69–1.15) and mean RHIE bout recovery (ES = 0.56). Inside and outside players also reported small to moderate differences (ES = 0.43–0.80) for times spent in each HR zone. There were a number of moderate to very large correlations between physical fitness measures and match-activity profile variables. This study found lower body muscular power, straight line running speed and Yo-Yo IR2 to be related to the match-activities of representative tag football players, although differences between inside and outside players suggest that athlete testing and training practices should be modified for different playing positions.

## Introduction

Tag football is an international game that is gaining rapid popularity around the world, particularly in Australia, New Zealand and the United Kingdom. The game is best described as a modified version of rugby league where players aim to remove Velcro tags attached to each player’s shorts rather than tackle their opponent. Elite-level competition is contested at state-, national- and international-representative levels and is played over 1–3 day tournament competitions. The rules of tag football vary slightly depending on which form of the game is played, however a typical match is contested by two teams of 16 players on a 70 m by 50 m playing field over two 20 min halves. There are only 8 players allowed on the field at any one time, although teams are allowed unlimited interchanges during a match. The unlimited interchange and modified contact laws promote a fast-paced running tempo and with the increasing competition standards of tag football, a better understanding of the physical and physiological demands of the game is becoming necessary to guide training practices.

The use of microtechnology devices incorporating global positioning system (GPS) and accelerometer technology have provided a useful tool to examine the match-activity profiles of team-sport athletes and guide sport-specific training practices [1–3]. Several recent studies have determined the match-activity profiles of tag football players during state- and national-level competition have shown the match-activity profile to be characterised by highly intermittent running efforts during short field rotations (3.5 to 4 min) that equate to average match work-rates comparable to professional rugby league players [4–8]. However, studies have yet to identify the fluctuations in work-rate or the most demanding passages of match-play where players may be required to perform successive high-intensity running efforts with minimal recovery. Previous research in similar team sports has shown that work-rate variables (e.g. m/min) can fluctuate considerably during a match and an understanding of the most demanding passages of play, although occurring infrequently, is required to adequately prepare players for competition [9, 10]. Several researchers have aimed to quantify the most demanding passages of play in team sports by determining the occurrence of repeated high-intensity effort (RHIE) bouts, defined as a minimum of three consecutive high-intensity running, maximal acceleration or physical collision efforts with less than 21 s between consecutive efforts [11, 12]. For example, an investigation by Spencer et al. [13] in elite field hockey showed players performed 30±14 sprinting efforts during a single match equating to approximately one sprint every 2 min. However, players also performed up to four repeated sprint bouts during the match, defined as a minimum of 3 sprint efforts with less than 21 s recovery, with several bouts consisting of up to six to seven consecutive efforts. These results demonstrate the importance of determining the most intense passages of match-play in order to prescribe training activities that adequately prepare players for competition.

Despite there being an increased research interest in the physical and physiological demands of tag football competition, there has been little attention given to the typical physical fitness profiles of players and the relationship between physical fitness and the match-activity profile in tag football. Physical fitness tests can provide a useful assessment of an athlete’s well-being, training progression and readiness to compete [14–16]. Several researchers in similar team sports such as soccer and rugby, have examined the relationship between physical fitness tests and players’ match-activity profile to determine which tests provide a useful assessment of sport-specific fitness [17–19]. For example, a study in youth soccer players demonstrated Yo-Yo intermittent recovery test level 1 (Yo-Yo IR1) to have strong associations with high-intensity running (r = 0.56; p<0.01) and sprinting (r = 0.63; p<0.01) distances during matches whilst maximal oxygen uptake (VO^2^max) determined during a laboratory treadmill test was found to have poor associations (r = 0.00; p = 0.99) with these measures [19]. Similarly, a recent investigation in tag football found players selected into the state-representative team had greater Yo-Yo intermittent recovery test level 2 (Yo-Yo IR2) performance than non-selected players in the same team, and Yo-Yo IR2 was associated with greater very high-intensity running (VHI) distance per minute (r = 0.77; p<0.01), VHI effort frequency (r = 0.75; p<0.01) and peak running speed (r = 0.77; p<0.01) during matches [7]. These studies demonstrate the usefulness of physical fitness tests to assess sport-specific fitness and provide information pertaining to an athlete’s game readiness and training progression in team sports.

Previous studies have provided important information on the match-activity profiles of representative tag football players at state- and national-level [4–7]. However, further research is required to determine the most demanding passages of match-play and the physiological responses of players to contribute to our understanding of the match-activity profile in tag football. There is also limited information on the typical physical fitness profiles of tag football players and the relation between physical fitness and match performance. Previous research in tag football has shown Yo-Yo IR2 performance to contribute to high-intensity running performance during tag football match-play, although it is uncertain whether this relationship is dependent upon playing position which has been shown to influence the match-activity profile [5, 7]. Gaining a better understanding of the relationship between physical fitness measures and the match-activity profile may have important implications for athlete testing and training practices in tag football. The purpose of this study was to a) examine the physical fitness, match-activity profiles and physiological responses of representative tag football players in relation to playing position and b) determine the relationship between physical fitness measures and the match-activity profile.

## Materials and Methods

### Study Design

This study employed a prospective and observational study design. Match-activity profiles and heart rate responses were analysed using microtechnology devices (MinimaxX S4, Catapult Sports, Melbourne, Australia) combined with heart rate (HR) chest straps (Polar, Krakow, Finland). Sixteen male players from the same team were analysed for five matches played over two consecutive days of competition. Match-activity profiles, heart rate responses and perceived match-intensities were examined based on players’ playing position (inside or outside players) and partial correlation coefficients were used to determine the relationship between selected match performance variables and physical fitness tests assessed prior to competition.

### Participants

Sixteen male tag football players from the same open men’s representative team participated in this study (Mean ± standard deviation (SD); age 24±4 yrs; height 1.78±0.06 m; body mass 75.9±9.4 kg). The participating team placed second out of sixteen teams during the 2014 Australian National Championships which is an annual tournament competed by regional-representative teams from Australia and New Zealand over three days of competition. It is the highest standard of tag football competition played in Australia and provides players with a pathway to represent Australia during the Triennial Tag Football World Cup. Players were informed of the study requirements prior to the tournament and all players and coaching staff gave their written informed consent to participate. All procedures were approved by the University of the Sunshine Coast’s Human Research Ethics Committee (HREC) in the spirit of the Declaration of Helsinki, HREC approval number S/13/471.

### Procedures

#### Preliminary fitness testing

Participants were required to complete a fitness test battery five days prior to the commencement of the Australian National Championships. The fitness test battery was performed before combined squad training following a two day unloading phase and prior to the team travelling interstate for the tournament. All players performed the tests at the same time of day on an outdoor playing surface and were instructed to maintain their usual diet prior to the commencement of training.

Lower body muscular power was assessed using a vertical jump test. Vertical jump height was determined using a yardstick vertical jump device (Swift Performance Equipment, Brisbane, Australia). Players were instructed to stand with feet flat on the ground with ankles in line with the yardstick, fully extend their inside arm and hand (dominant), and mark the standing reach height. Players where then instructed to perform a rapid countermovement jump from a standing start and touch the highest point possible. Vertical jump height was calculated as the distance from the highest point reached minus the initial starting height following three maximal attempts. The vertical jump test was shown to have acceptable reliability in this study’s participant cohort (CV = 3.3%; ICC = 0.95).

Straight line running speed was assessed using dual beam electronic timing gates (Fusion Sport, Sumner Park, Australia) with 10 m and 20 m splits. Players started 30 cm behind the first timing gates placed at 0 m and instructed to run as quickly as possible along the 20 m distance from an athletic start. Speed was measured to the nearest 0.01 s with the fastest value over 20 m used as the score. Straight line running speed measures had acceptable reliability over 10 m (CV = 1.9%; ICC = 0.92) and 20 m (CV = 1.5%; ICC = 0.96) in this study’s participant cohort.

Yo-Yo intermittent recovery test level 2 (Yo-Yo IR2) was performed on an outdoor playing surface following the vertical jump and straight line running speed assessment. The procedures of the test have been described in detail elsewhere [20]. All players had been familiarised with the testing procedures of the Yo-Yo IR2 during previous training sessions. The Yo-Yo IR2 was shown to have acceptable reliability within this study’s participant cohort (CV = 6.6%; ICC = 0.96).

#### Match-activity profiles

Micortechnology devices worn by players during matches provided GPS data sampling at 10 Hz (MinimaxX S4, Catapult Sports, Melbourne, Australia). A total of 80 match-files were collected over five matches played by the team during two days of competition. However, 9 incomplete match-files were discarded from analysis due to battery failure or player injury during match-play resulting in a total of 3–5 match-files per player being included in data analysis. The microtechnology device was worn in a small custom made vest in between the shoulder blades on the upper back of each player. Players were familiar with the device and the custom made vest which they had worn on numerous occasions prior to the tournament. The device was switched on and locked to satellites prior to the team warm-up. Recorded data was compiled and analysed in Sprint 5.1 (Catapult Sports, Melbourne, Australia). Periods of time where players were off the field, including interchanges and half-time periods, were excluded from data analysis.

This study categorised GPS data into low-speed running (LSR = 0.4–8.0 km/h), moderate-speed running (MSR = 8.1–14.0 km/h), high-speed running (HSR = 14.1–18.0 km/h) and very high-speed running (VHSR = >18.1 km/h). These speed zones were assigned as they have previously been used to describe match-activity profiles in tag football [4–7, 21] and the microtechnology device used in this study has been shown to provide accurate and reliable distance and effort measurements at velocities ranging from 0–14 km/h and 14–20 km/h during stimulated team sport running patterns [22, 23]. High acceleration and deceleration efforts >2.78 m/s^2^ and lasting a minimum of 0.4 s were also recorded. The microtechnology device used in this study has been shown to detect instantaneous changes in velocity during acceleration with a percentage bias of -3.6% to -2.1% and a typical error of 2.6% to 5.9% against a criterion measure of a laser [24]. Occurrences of acceleration and deceleration efforts were only recorded if they lasted a minimum of 0.4 s as this method has been shown to improve the ecological validity of acceleration measures derived from GPS [25]. The RHIE bouts performed by of players were recorded as a minimum of three high-speed running (>14 km/h) or high acceleration efforts (>2.78 m/s^2^) performed with less than 21 s recovery between consecutive efforts as similarly recommend for other team sports [8, 12].

Mean and peak match heart rates (HR) were expressed relative to players’ peak HR (%HRpeak), determined during the Yo-Yo IR2 (HRpeak = 197±8 bpm). As previously employed in similar team sports [26, 27], HR data were categorised based on the percentage of on-field time spent in each of the following HR zones: Zone 1 (<60% HRpeak), Zone 2 (61–70% HRpeak), Zone 3 (71–80% HRpeak), Zone 4 (81–90% HRpeak), Zone 5 (91–95% HRpeak) and Zone 6 (>95% HRpeak). Players’ rating of perceived exertion (RPE) for matches was obtained using Borg’s CR-10 RPE scale [28]. Players provided there scores individually, so that their scores were blinded from other team mates, approximately 10–15 min following matches. The RPE score was then multiplied by players’ on-field playing time to provide a measure of internal load (Session-RPE, AU) [28]. Players were familiarised with the Borg’s CR-10 RPE scale to assess perceptual match and training intensity prior to the commencement of the tournament.

### Statistical Analyses

Data was log transformed prior to analysis to reduce non-uniformity of error and back transformed to attain descriptive statistics. Standardised differences were calculated and classified as trivial (<0.2), small (0.2–0.6), moderate (0.6–1.2), large (1.2–2.0) and very large (>2.0) as per previously standardised criteria [29]. When the 90% confidence limit (CL) crossed the threshold for both substantially positive (0.2) and negative (-0.2) values the difference was reported as unclear [29]. Pearson’s correlation coefficients were calculated to determine the relationship between physical fitness measures and mean and peak match-activity measures for the combined group (n = 16) and inside (n = 8) and outside (n = 8) playing positions. Correlations are reported as trivial (<0.1), small (0.1–3.0), moderate (0.3–0.5), large (0.5–0.7), very large (0.7–0.9) and almost perfect (>0.9) [30]. When the 90% CL crossed the threshold for both a substantially positive (0.1) and negative (0.2) association the correlation was determined as unclear [30].

## Results

The physical fitness scores for players based on playing position are presented in Table 1. There were moderate differences between inside and outside playing positions for vertical jump and measures of straight line running speed. There was no difference in Yo-Yo IR2 scores between playing positions.

**Table 1:** Physical fitness of representative tag football players based on playing position.

	Combined (n = 16)	Inside players (n = 8)	Outside players (n = 8)	p value	ES±90% CI	QO
**Age (yrs)**	24 ± 4	26 ± 3	22 ± 2	<0.01	1.25 ± 0.71	Large
**Body mass (kg)**	75.9 ± 9.4	76.7 ± 9.7	75.2 ± 9.7	0.76	0.16 ± 0.91	Unclear
**Height (m)**	1.78 ± 0.06	1.75 ± 0.03	1.81 ± 0.07	0.04	1.05 ± 0.79	Moderate
**Vertical jump (cm)**	53.7 ± 5.0	51.3 ± 3.2	56.1 ± 5.4	0.05	0.98 ± 0.80	Moderate
**10 m speed (s)**	1.65 ± 0.08	1.69 ± 0.08	1.60 ± 0.07	0.04	1.02 ± 0.78	Moderate
**20 m speed (s)**	2.91 ± 0.12	2.98 ± 0.09	2.84 ± 0.10	0.01	1.18 ± 0.72	Moderate
**0–10 m velocity (km/h)**	21.9 ± 1.1	21.4 ± 1.0	22.5 ± 1.0	0.03	1.03 ± 0.77	Moderate
**10–20 m velocity (km/h)**	28.5 ± 1.1	27.9 ± 0.9	29.1 ± 1.0	0.02	1.08 ± 0.76	Moderate
**Yo-Yo IR2 (m)**	1023 ± 346	1115 ± 406	930 ± 269	0.30	0.53 ± 0.89	Unclear

Data are mean ± standard deviation (SD). ES = standardised difference. CI = confidence interval. QO = qualitative outcome. Yo-Yo IR2 = Yo-Yo intermittent recovery test level 2.

**Fig 1:**
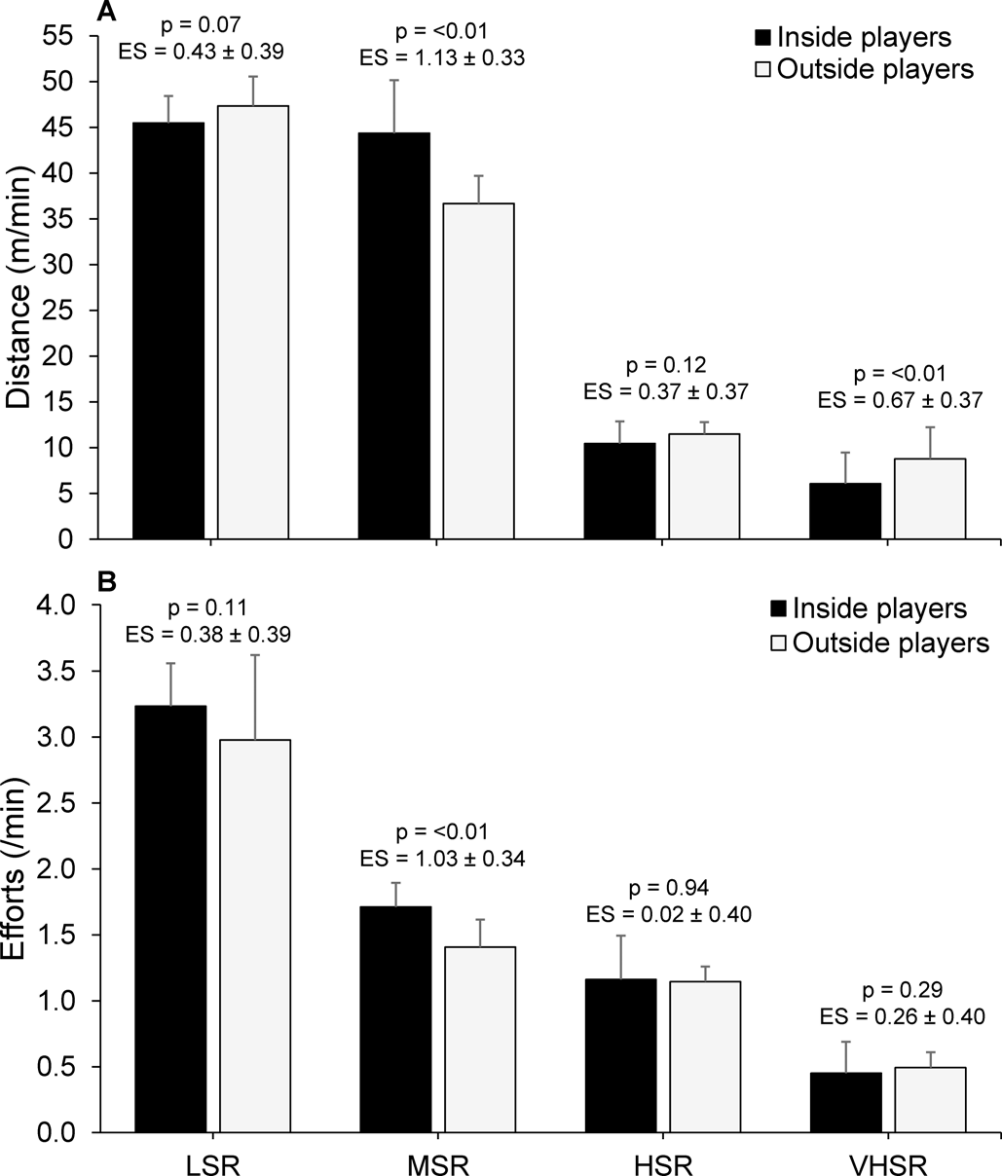
Distance covered per minute (A) and effort frequency (B) of tag football players in low- (LSR), moderate- (MSR), high- (HSR) and very high-speed running (VHSR) velocities based on playing position.

Table 2 presents the activity profiles of tag football players and differences based on playing position. There were moderate differences between inside and outside players for peak running speed (ES = 0.95), mean and maximal VHSR effort distances (ES = 0.84–0.86), and maximal acceleration and deceleration frequency (ES = 0.60–0.80). Although there were only small differences in distance/min between playing positions, outside players covered greater VHSR m/min and less MSR m/min compared to inside players (Fig 1A). Inside and outside players reported similar effort frequencies at different running speeds, except inside players performed more MSR efforts/min (Fig 1B).

**Table 2:** Match-activity profiles and physiological responses of representative tag football players based on playing position.

	Combined (n = 71)	Inside players (n = 35)	Outside players (n = 36)	p value	ES±90% CI	QO
**Field time (min)**	20.7 ± 2.4	20.7 ± 1.0	20.8 ± 1.4	0.95	0.01 ± 0.40	Unclear
**Total distance (m)**	2176 ± 258	2198 ± 121	2153 ± 163	0.40	0.20 ± 0.40	Small
**Distance (m/min)**	105.5 ± 9.1	106.4 ± 7.4	104.3 ± 5.6	0.27	0.26 ± 0.39	Small
**Peak running speed (km/h)**	26.6 ± 2.8	24.8 ± 1.6	28.3 ± 2.7	<0.01	0.95 ± 0.35	Moderate
**Mean VHSR effort distance (m)**	15.4 ± 4.4	13.5 ± 1.7	17.1 ± 3.4	<0.01	0.84 ± 0.36	Moderate
**Maximal VHSR effort distance (m)**	34.1 ± 13.7	28.4 ± 5.8	39.4 ± 10.3	<0.01	0.86 ± 0.36	Moderate
**High accelerations (/min)**	0.83 ± 0.28	0.93 ± 0.29	0.74 ± 0.11	0.01	0.60 ± 0.38	Moderate
**High decelerations (/min)**	1.13 ± 0.37	1.29 ± 0.38	0.98 ± 0.11	<0.01	0.80 ± 0.37	Moderate

Data are mean ± standard deviation (SD). ES = standardised difference. CI = confidence interval. QO = qualitative outcome. VHSR = very high-speed running.

The RHIE characteristics of players based on playing position are presented in Table 3. Inside and outside players reported a similar number of total RHIE bouts and mean and maximum efforts per RHIE bout. However, there were small to moderate differences for mean and maximum effort durations (ES = 0.69–1.15) and mean bout recovery (ES = 0.56).

**Table 3:** Repeated high-intensity effort (RHIE) characteristics of representative tag football players based on playing position.

	Combined (n = 71)	Inside players (n = 35)	Outside players (n = 36)	p value	ES±90% CI	QO
**RHIE total bouts (no.)**	6.8 ± 2.6	6.9 ± 2.7	6.7 ± 1.2	0.90	0.03 ± 0.40	Unclear
**Mean efforts per bout (no.)**	4.1 ± 0.6	4.1 ± 0.6	4.1 ± 0.3	0.75	0.08 ± 0.40	Unclear
**Maximum efforts per bout (no.)**	6.6 ± 2.7	6.9 ± 2.8	6.4 ± 1.0	0.56	0.14 ± 0.40	Unclear
**Mean effort duration (s)**	1.6 ± 0.4	1.4 ± 0.1	1.8 ± 0.2	<0.01	1.15 ± 0.32	Moderate
**Maximal effort duration (s)**	5.6 ± 1.8	5.1 ± 1.4	6.2 ± 1.1	<0.01	0.69 ± 0.37	Moderate
**Mean effort recovery (s)**	6.2 ± 1.1	6.3 ± 0.5	6.0 ± 0.8	0.37	0.20 ± 0.40	Unclear
**Mean bout recovery (s)**	62.9 ± 55.8	51.0 ± 20.0	74.2 ± 21.1	0.02	0.56 ± 0.38	Small

Data are mean ± standard deviation (SD). ES = standardised difference. CI = confidence interval. QO = qualitative outcome. RHIE = repeated high-intensity effort.

**Fig 2:**
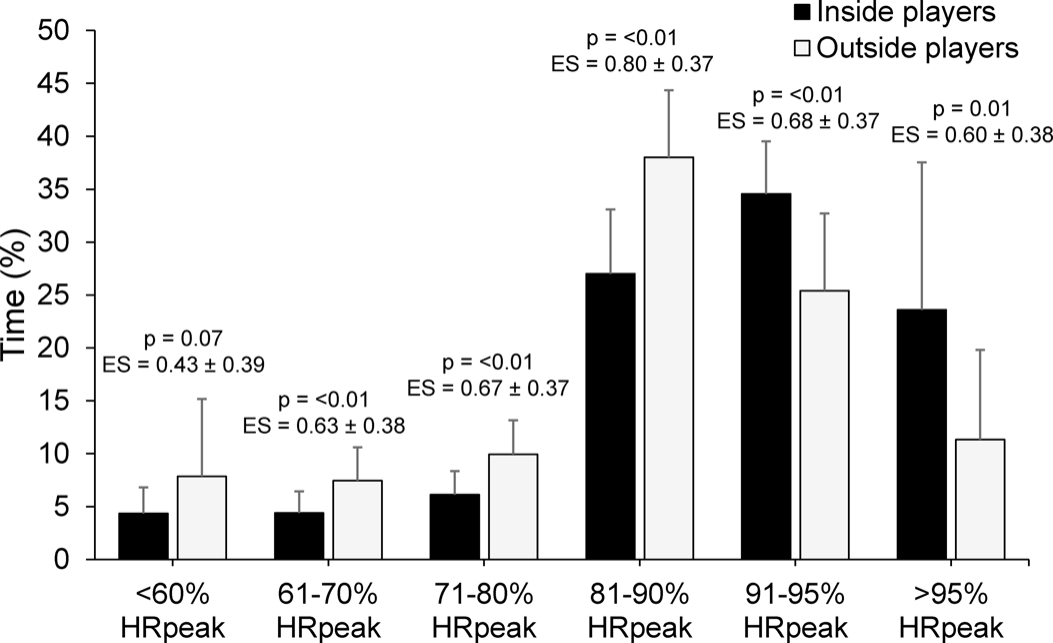
Percentage of time spent in various heart rate zones for tag football players based on playing position.

Players’ perceived match-intensities and heart rate responses are presented in Table 4. Inside and outside players reported no differences in RPE or Session-RPE for matches, however there was a moderate difference in mean HR (ES = 0.67) between playing positions. There were also differences in the distribution of time spent at various heart rates between playing positions (Fig 2), with inside players spending a greater percentage of time at heart rates above 90% HRpeak compared to outside players spending a greater percentage of time at heart rates between <60–90% HRpeak.

**Table 4:** Physiological responses of representative tag football players based on playing position.

	Combined (n = 71)	Inside players (n = 35)	Outside players (n = 36)	p value	ES±90% CI	QO
**RPE (au)**	4.7 ± 1.4	4.6 ± 0.8	4.8 ± 0.6	0.81	0.06 ± 0.40	Unclear
**Session-RPE (au)**	96.5 ± 29.3	95.6 ± 15.5	98.6 ± 13.2	0.71	0.09 ± 0.40	Unclear
**Mean match HR (bpm)**	170 ± 13	173 ± 12	168 ± 12	0.09	0.40 ± 0.39	Small
**Mean match HR (% HRpeak)**	86.4 ± 5.0	88.3 ± 2.4	84.9 ± 3.6	<0.01	0.67 ± 0.37	Moderate
**Peak match HR (bpm)**	192 ± 9	192 ± 10	192 ± 8	0.96	0.01 ± 0.40	Unclear
**Peak match HR (% HRpeak)**	97.8 ± 3.1	98.1 ± 1.2	97.2 ± 2.6	0.19	0.31 ± 0.39	Small

Data are mean ± standard deviation (SD). ES = standardised difference. CI = confidence interval. QO = qualitative outcome. RPE = rating of perceived exertion. HR = heart rate. HRpeak = peak heart rate determined during the Yo-Yo IR2.

**Fig 3:**
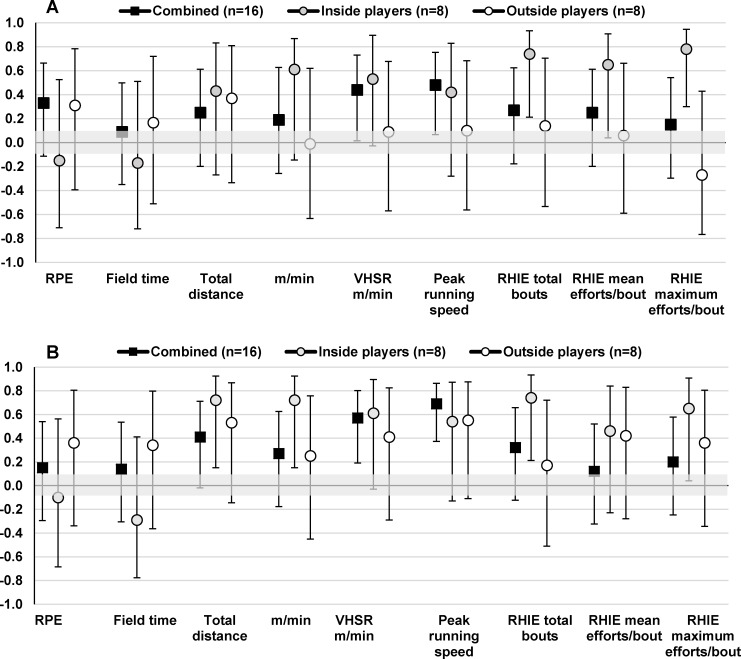
Pearson correlation coefficients (±90% CI) describing the relationship between vertical jump and mean (A) and peak (B) match performance measures for the combined group and inside and outside playing positions.

**Fig 4:**
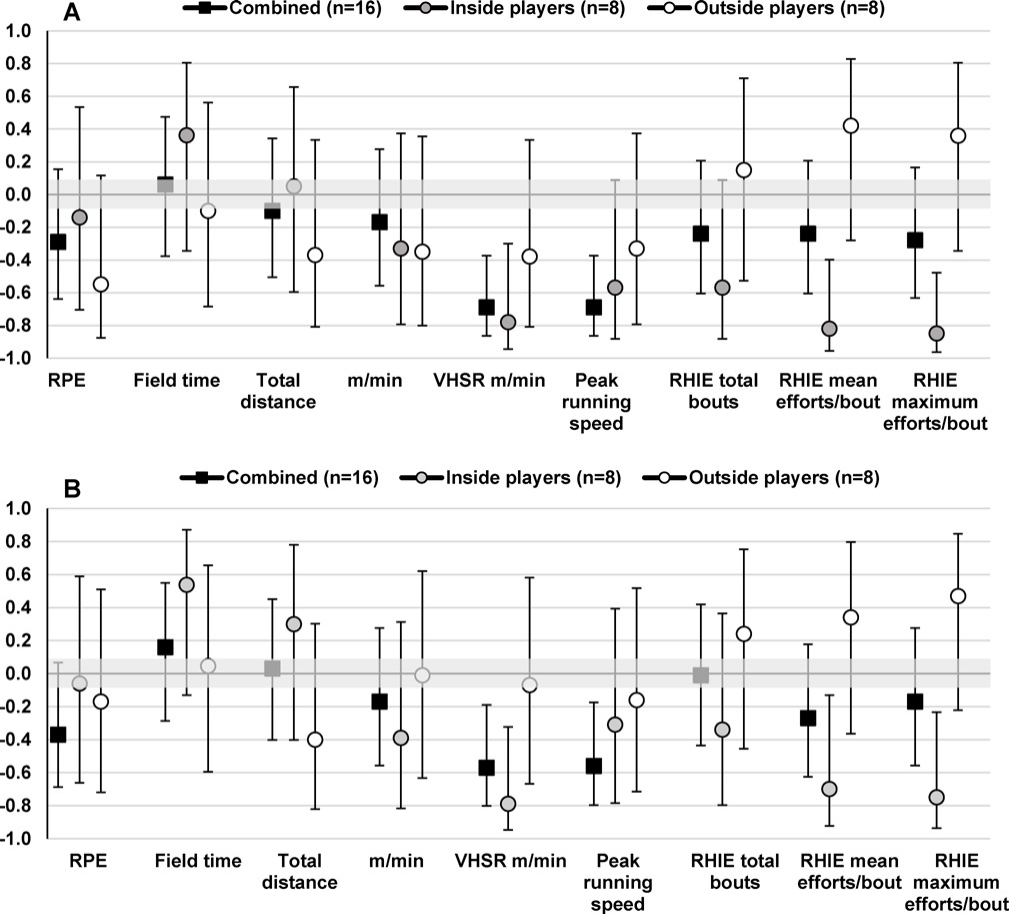
Pearson correlation coefficients (±90% CI) describing the relationship between 20 m speed and mean (A) and peak (B) match performance measures for the combined group and inside and outside playing positions.

**Fig 5:**
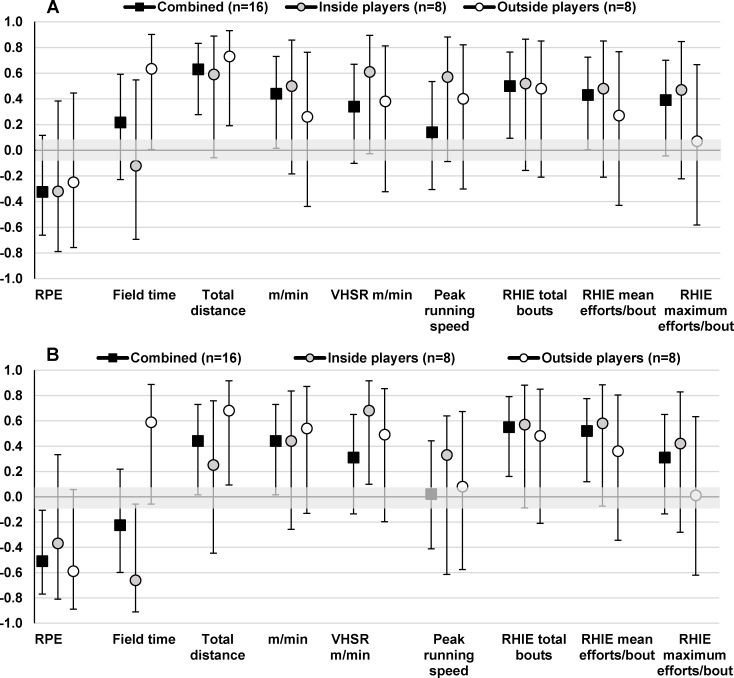
Pearson correlation coefficients (±90% CI) describing the relationship between Yo-Yo IR2 and mean (A) and peak (B) match performance measures for the combined group and inside and outside playing positions.

Vertical jump and straight line running speed (20 m speed) showed moderate to large correlations with VHSR m/min (r = 0.44–0.69), VHSR efforts/min (r = 0.44–0.50) and peak running speeds (r = 0.48–0.69) for the combined group (Fig 3 and Fig 4). Inside players (r = 0.54–0.81) showed stronger correlations between vertical jump and straight line running speed measures and VHSR m/min, VHSR efforts/min and peak running speeds than outside players (r = 0.05–0.55). Further, vertical jump and straight line running speed measures showed moderate to very large correlations with RHIE total bouts for inside players (r = 0.46–0.85) and weaker correlations with outside players (r = 0.01–0.49). Yo-Yo IR2 was positively correlated with total distance (r = 0.59–0.73), relative distance (0.26–0.50), VHSR m/min (r = 0.38–0.61), VHSR efforts/min (r = 0.55–0.58), peak running speeds (0.40–0.56), RHIE total bouts (r = 0.48–0.52), and mean efforts per RHIE bout (r = 0.27–0.48) for inside and outside players whilst showing negative correlations with match RPE (r = -0.25 to -0.31) (Fig 5).

## Discussion

This study is the first to determine the normative data on a range of physical fitness tests in representative tag football players and provides coaching and support staff with useful information for designing training programs. Firstly, this study found vertical jump, straight line running speed and Yo-Yo IR2 performance to be associated with the match activities of tag football players suggesting that these tests may provide a useful assessment of a player’s well-being, training progression and readiness to compete. Importantly though, there were a number of differences in both physical fitness and match-activity profiles between inside and outside playing positions. For greatest transference to match performance, training programs for tag football players should be individualised based on playing position to prioritise the development of certain physical fitness qualities. The RHIE characteristics identified in this study may be used by coaches to design sport-specific fitness drills that replicate the most intense passages of tag football match-play and modify certain training parameters to promote positional-specific training adaptations.

The current study found a number of differences in physical fitness measures between playing positions. Outside players were found to have greater lower body muscular power and faster straight line running speed than inside players. This finding can likely be attributed to the different positional-specific demands of inside and outside players in tag football. For instance, outside players were found to have greater mean and maximal VHSR effort distances and reach higher peak running speeds than inside players (Table 1) and the ability to produce higher peak running speeds during matches may be more beneficial for players positioned towards the edges of the field where there is typically more space between them and their opposing player [5, 31–33]. Further, outside players likely have a greater chance to recover between repeated VHSR efforts compared to inside players, as evident through differences in high acceleration and deceleration frequencies (Table 2), MSR effort frequency (Fig 1B), and time spent above 90% HRpeak (Fig 2) between inside and outside playing positions [34, 35]. Although outside players exhibited faster straight line running speed, it is interesting to note that inside players showed stronger correlations between straight line running speed and VHSR m/min, VHSR effort frequency and peak running speed than outside players (Fig 4). These results may be explained by the test distance of 20 m, as outside players had a maximal VHSR effort distance of 39±10 m. Therefore, the ability of outside players to produce higher running velocities between 20–40 m distances may have stronger correlations with their match-activity profile, whilst the ability to produce faster running speeds between 10–20 m is more beneficial for inside players [5, 34, 36]. Further research should examine straight line running speed over short distances (10–20 m) as well as longer distances more typical of the maximal sprint distance (≥40 m) in tag football to provide a better understanding of the relationship between straight line running speed and match running performance for different playing positions.

This study found the Yo-Yo IR2 to be a useful determinant of high-intensity running performance during tag football match-play (Fig 5). The combined group showed moderate to large correlations between Yo-Yo IR2 test distance and match-activity profile variables including total distance (r = 0.44–0.63), VHSR m/min (r = 0.31–0.34), VHSR effort frequency (r = 0.44–0.50) and RHIE total bouts (r = 0.50–0.55). This is in agreement with previous research in tag football that found the Yo-Yo IR2 to be a useful determinant of high-intensity running performance at state-level competition [7]. In the current study, inside players showed stronger correlations between Yo-Yo IR2 and distance/min (r = 0.50 vs. r = 0.26), VHSR m/min (r = 0.61 vs. r = 0.31), RHIE mean efforts per bout (r = 0.48 vs. r = 0.27) and RHIE maximum efforts per bout (r = 0.47 vs. r = 0.07) than outside players, suggesting that the Yo-Yo IR2 may be more beneficial to the match-activities of inside players. Indeed, higher Yo-Yo IR2 may allow players to better maintain high-intensity running performance in-between more frequent high acceleration and deceleration efforts (Table 2) and MSR efforts (Fig 1B) as characteristic of the match-activity profile of inside players [20, 35]. There were also moderate differences between inside and outside playing positions for the amount of time spent >90% HRpeak suggesting that inside players may require higher aerobic and anaerobic capacities than outside players due to their positional-specific demands (Fig 2) [35, 37]. Therefore, although Yo-Yo IR2 appears to be related to the match-activities of both inside and outside players, the results of this study suggest that training programs should be adjusted based on playing position to prioritise the development of certain physical fitness qualities. It is recommended that training programs for inside players prioritise the development of physical fitness qualities related to the Yo-Yo IR2 whilst training programs for outside players have a greater focus on developing lower body muscular power and straight line running speed.

This study is the first to determine the RHIE characteristics of tag football and can be used to guide positional-specific conditioning practices for tag football players. Generally, there were no differences in the number of efforts performed for bouts or the mean recovery between consecutive efforts based on playing position (Table 3). Therefore, it is recommended that RHIE training drills for tag football players incorporate 4–8 repeated efforts with approximately 6 s recovery between efforts. There were differences in the mean and maximal effort durations between inside and outside playing positions, with outside players typically having longer mean and maximal effort durations in line with the greater VHSR effort distances performed by these players. Therefore, RHIE training drills may be modified for positional groups, with outside players covering 15–40 m effort distances and inside players covering 10–30 m effort distances to mimic their positional-specific demands. Further, to allow outside players to comply with the increased high-intensity running performed during RHIE drills due to greater effort distances, outside players should have greater recovery between bouts (75±20 s) compared to inside players (50±20 s) [35]. Although the RHIE characteristics determined in this study can be used to guide sport-specific conditioning practices in tag football, it should be recognised that not all conditioning needs to be sport-specific. Due to the fatiguing nature of this type of exercise, high volumes of training replicating the RHIE characteristics of tag football match-play may lead to adverse training adaptations such as injury, illness or overtraining [38]. Further, previous research in similar team sports has shown measures of aerobic and anaerobic fitness to be positively related to payers’ repeated-sprint ability [39]. It is recommended that the inclusion of training drills replicating the RHIE characteristics of tag football match-play is appropriately planned based on the amount of high-intensity running that players are performing during other training and competition activities and following the development of aerobic and anaerobic capacities.

Despite tag football being a recreational sport, players competing at elite-representative levels appear to have comparable physical fitness qualities than professional and semi-professional athletes of similar team sports. For example, in the current study tag football players reported slightly faster 10 m sprint times (1.65±0.08 s) than international rugby sevens players (1.68–1.74 s) and professional rugby league players (1.66–1.72 s); and covered distances in the Yo-Yo IR2 (1023±346 m) comparable to professional Australian football players (1028±190 m) and greater than first division recreational Australian football players (880±260 m) [15, 40–43]. Although caution should be taken when making comparisons between these studies due to differences in testing conditions, the results of this study provide interesting normative data from a single team and suggest representative tag football players competing at national-level exhibit high levels of physical fitness. Previous research in tag football showed players from the same team competing at state-level competition covered 892±170 m during the Yo-Yo IR2 under similar testing conditions than the current study [7]. This study also showed that players selected into the state-representative team covered greater distances during the Yo-Yo IR2 than non-selected players suggesting that the Yo-Yo IR2 may be a useful test to assess player capabilities and select playing rosters in tag football. Similarly, the higher Yo-Yo IR2 scores shown for national-level players in the current study compared to state-level players (1023±346 m vs. 892±170 m) suggests that there may be a relation between higher Yo-Yo IR2 and playing standard attained in tag football.

Whilst this study provides several important findings for coaching and support staff working with tag football players, there are a number of limitations that warrant discussion. Firstly, this study included data that was from a single team and it is possible that the results of this study may not be evident in other teams competing at national-level due to differences in team dynamics and playing strategies. Further, this study collected limited data on the physical fitness qualities of players due to restrictions applied to the scheduling and time allocation of performance testing. We reconciled these limitations with the knowledge that the participating team was of a high-standard, finishing second out of sixteen teams at the conclusion of the national championships. This study was also the first to determine the relationship between physical fitness measures and the match-activity profiles of tag football players competing at national-level and provides important information for the development of future research studies. Future studies that include larger sample sizes from different competition standards and examine the relevance of different physical fitness tests (e.g. Yo-Yo IR2 vs. 30–15 IRT) may allow for further developments in athlete training and testing practices in tag football.

## Conclusions

This study provides important information for coaching and support staff working with tag football players. Firstly, this study provides the first normative data for a range of physical fitness measures for male tag football players competing at national-level. Lower body muscular power, straight line running speed and Yo-Yo IR2 were found to be important physical fitness qualities related to the match activities of both inside and outside players. Therefore, these tests provide a useful assessment of players’ physical fitness and may be used to evaluate the effectiveness of training in tag football. Coaching and support staff working in tag football may use this data to identify key areas of physical fitness that a player or group of players may need to develop based on their playing position. The results of this study suggest that training programs for inside players should have a greater focus on the development of physical capacities related to the Yo-Yo IR2 whilst outside players should focus on developing lower body muscular power and straight line running speed over short distances (10–50 m).

The results of this study may also be used to guide sport-specific conditioning drills for tag football players. Based on the RHIE characteristics of players’ match-activity profile it is recommended that repeated-sprint drills incorporate 4–8 repeated efforts with approximately 6 s recovery between efforts. Repeated-sprint drills may be modified for positional groups by adjusting effort distances and bout recovery. Inside players should perform 10–30 m efforts and have 50±20 s of recovery between consecutive bouts. Outside players should perform consecutive efforts over longer distances (15–50 m) whilst having longer recovery between consecutive bouts (75±20 s) to mimic their positional-specific demands. Importantly, the volume of this type of training should be adjusted during certain training phases following appropriate planning and the development of aerobic and anaerobic capacities to avoid increases in the risk of injury, illness and overtraining.
